# Knowledge and attitudes of pregnant women about COVID-19 vaccination

**DOI:** 10.1590/1518-8345.7331.4521

**Published:** 2025-05-02

**Authors:** Serap Tekbaş

**Affiliations:** 1 Izmir Tinaztepe University, Faculty of Health Sciences, İzmir, Turkey

**Keywords:** Vaccine, COVID-19, Women, Pregnancy, Knowledge, Attitude

## Abstract

to assess the knowledge and attitudes of pregnant women towards COVID-19 vaccination and the factors that affect them.

this is a cross-sectional and analytical study with 407 pregnant women. The COVID-19 Vaccine Knowledge and Attitude Scale was used to assess the knowledge and attitudes of pregnant women towards COVID-19 vaccination. Mann-Whitney U and Kruskal-Wallis H tests were used for statistical analysis.

it was found that 63.88% of the sample had a negative opinion about being vaccinated against COVID-19 during pregnancy. Seventy-five percent of the pregnant women believed that the vaccine would harm their fetus. The mean subscale scores of the severity (*p* = 0.001) and benefit (*p* = 0.001) of the vaccine were significantly higher in pregnant women with a higher level of education and in the first trimester (*p* = 0.001). It was found that pregnant women who stated that they had sufficient information about COVID-19 had significantly higher severity (*p* = 0.001) and benefit (*p* = 0.031) subscale mean scores and had a more positive attitude against COVID-19 vaccination during pregnancy.

most pregnant women had negative attitudes due to concerns that the COVID-19 vaccine would harm the fetus. Healthcare professionals should provide education about the importance of vaccination during pregnancy for maternal and infant health during pregnancy follow-up.

## Highlights

**(1)** The study evaluated vaccine acceptance and COVID-19 vaccine attitude during pregnancy. **(2)** Pregnant women participated in the study. **(3)** Negative attitudes towards vaccines during pregnancy was detected. **(4)** The study results can be a guide to inform health institutions about vaccine acceptance.

## Introduction

COVID-19, caused by severe acute respiratory syndrome coronavirus 2 (SARS-CoV-2), has caused massive morbidity and mortality globally^1^. From 2019, when the first coronavirus cases were detected, to July 2024, more than 775 million confirmed cases and more than 7 million deaths were reported worldwide. In addition, approximately 14 billion doses of COVID-19 vaccine have been administered^2^.

Due to physiological and immunological changes that occur during pregnancy, pregnant women become more susceptible to respiratory tract infections, putting them at high risk for serious outcomes in the event of COVID-19^3^.

Studies have reported that COVID-19 infection worsens the clinical course in pregnant women compared to non-pregnant women of the same age^4^. The US Centers for Disease Control and Prevention (CDC) reports that pregnant women are three times more likely to be admitted to the intensive care unit or require intubation and 1.5 times more likely to die from COVID-19 than non-pregnant women^5^.

Adverse effects of COVID-19 have also been found in terms of fetal health and development. In a meta-analysis study, it was found that pregnant women with SARS-CoV-2 infection had a higher risk of stillbirth and preterm birth than pregnant women without the infection^6-7^. Moreover, SARS-CoV-2 infection can trigger a cytokine storm, which can lead to both an inflammatory response in the fetus and placental damage with subsequent fetal growth restriction, preterm labor, and miscarriage^8^.

Many factors affect individuals’ attitudes towards vaccination. These factors include the type of vaccine, geography, culture, and socioeconomic status. People may also have reservations about accepting new vaccines. Hesitancy about vaccination may increase during pregnancy due to concerns about its effects on the fetus^9-10^.

One of the most effective approaches against COVID-19, which causes severe acute respiratory syndrome, is vaccination^10-11^. Because none of the COVID-19 vaccines contain live viruses or adjuvants that could harm an unborn child, several studies, the American College of Obstetricians and Gynecologists^(5,12-14)^ and the Society for Maternal-Fetal Medicine recommend vaccination for pregnant and breastfeeding women^15-16^. While the American College of Obstetricians and Gynecologists recommends the COVID-19 vaccine to all pregnant women, it has been stated that the decision should be left to the woman after individual risk factors are carefully assessed^5^. The literature emphasizes that vaccine-related reactions in pregnant and breastfeeding individuals are similar to that of the general population. In addition, research results report that vaccinated pregnant individuals are less likely to contract COVID-19 and that the disease has a milder course in infants born to vaccinated mothers^17^. Pregnant women generally have lower willingness and greater concern about vaccination against vaccine-preventable diseases (e.g., influenza and tetanus) than the general population^10^. It has been determined that women who have a negative attitude towards vaccines during pregnancy also have vaccine hesitancy towards the COVID-19 vaccine^4^. This situation shows that developing a positive attitude towards vaccination during pregnancy can reduce vaccine hesitancy towards the COVID-19 vaccine and potentially new vaccines^18-20^.

Given the increased morbidity associated with COVID-19 in pregnancy, it is important to determine pregnant women’s knowledge and attitudes towards COVID-19 vaccination during pregnancy in order to protect the pregnant woman and fetus from COVID-19 and reduce the adverse effects of the disease on pregnancy and the fetus^21-22^. For these reasons, this study was planned to evaluate the knowledge and attitudes of pregnant women toward the COVID-19 vaccine and the factors affecting their knowledge and attitudes.

## Method

### Type of study

This research is a descriptive cross-sectional, and analytical study. It is based on the Strengthening the Reporting of Observational Studies in Epidemiology (STROBE) guidelines.

### Study location, and population

The study was conducted from January to April 2022 in Northern Cyprus, which is a 9,251 km2 island in the Mediterranean where tourism is widespread and there are many students enrolled into 22 universities. For these reasons, the students population as academics as well as for working purposes is quite high. In addition, Northern Cyprus is an island with a very low birth rate and according to official data, the total number of births in the country in 2012 was 3,614^24^. Deliveries of women who have immigrant status for education and work purposes were also included in this number.

Pregnant women over the age of 18 who voluntarily participated in the study were included in the study, regardless of their gestational age. Data was collected online to prevent the risk of infection due to the pandemic. The surveys were designed by researchers using Google Forms. The link to the created Google Form was shared by the researcher on social media platforms (WhatsApp, Messenger, Facebook, and Instagram). The first page of the online questionnaire included information about the purpose and content of the study and the informed consent form for participation in the study. The form was created in a way that those who agreed to participate went on with the questionnaire.

### Sample selection

The number of pregnant women to be included in the sample was determined by the power analysis method. The birth rate statistics of the country were used as a reference for statistical power analysis^23^. The number of pregnant women required for the study was found to be 365 with α=0.05, 1-β=0.80, and 0.20 error rate. Of the women invited to the study, 407 accepted to participate and constituted the sample group of the study. After the study was completed, post-hoc power analysis was performed to determine the adequacy of the sample size. According to the power analysis, it was determined that the total sample was sufficient with an effect size of 0.92, 99% power, and 0.05% margin of error.

### Inclusion criteria

Pregnant women aged 18 years and over, without communication barriers (not speaking Turkish, mental health issues etc.), literate, with basic digital literacy were included in the study.

### Exclusion criteria

Pregnant women with any risk factors during pregnancy (e.g., pre-eclampsia, intrauterine growth retardation, premature rupture of membranes, gestational diabetes etc.) and pregnant women with any diagnosed problems related to the fetus’ health (e.g., fetal anomaly, intrauterine growth retardation) were excluded.

### Data collection instruments

The first 14 items of the form with 27 questions prepared by the researcher based on the literature review were rated to determine the socio-demographic characteristics of the participants^(20,24)^. Thirteen questions included the vaccination status of the participants, their views on getting COVID-19 vaccine during pregnancy, and vaccine information sources^(1,7,24-25)^.

#### COVID-19 Vaccine Knowledge and Attitude Scale

The scale, which was developed in 2021 to evaluate the Knowledge and Attitude of individuals concerning the COVID-19 vaccine, is a five-point Likert-type scale and consists of 16 questions^26^. The scale has no cut-off point. The high mean score obtained in each subscale indicates that the perceptions regarding seriousness, obstacle, and benefit subscale are high. The scale consists of a total of three subscales: “Perception of Severity of COVID-19 Disease” (5 items), “Perception of Vaccine Barriers” (7 items), and “Perception of Vaccine Benefit” (4 items). In scale scoring, each subscale is scored in itself. While high scores on the severity and benefit subscales indicate a positive attitude toward COVID-19, a high score on the barrier subscale indicates a negative attitude toward COVID-19. The Cronbach alpha coefficient of the scale used in our study was calculated as 0.83.

### Data analysis

In the study, the socio-demographic characteristics and vaccination status of the pregnant women and their distribution according to some characteristics related to COVID-19 were determined by frequency analysis. Descriptive statistics regarding the COVID-19 Vaccine Knowledge and Attitude Scale scores of the pregnant women were given and compliance with the normal distribution was examined with the Kolmogorov-Smirnov test. Nonparametric hypothesis tests such as Mann-Whitney U and Kruskal-Wallis H tests were used in the study, because the COVID-19 Vaccine Knowledge and Attitude Scale scores of pregnant women did not show a normal distribution.

### Ethical aspects

This study was approved by the University’s Institutional Review Board (nº 2021/98-1444). Before starting the survey, the consent of all participants was obtained, and the confidentiality of personal information was ensured. The study was conducted according to the ethical principles of the Declaration of Helsinki.

## Results

Table 1 presents the socio-demographic characteristics of the study conducted with the participation of 407 pregnant women. The mean age of the pregnant women participating in the study was 27.61±4.73 years old. It was determined that 27.27% of the participants were in the 1^st^ trimester, 40.05% were in the 2^nd^ trimester and 32.68% were in the 3^rd^ trimester. A fraction of 62.41% was in their first pregnancy, 28.99% was in their second pregnancy, and 28.26% already had a child.

It was determined that 13.51% of the pregnant women were secondary school graduates, 18.43% were high school graduates and 52.83% were university graduates. Circa two-thirds, 75.43%, had an income equal to expenses, 71.74% were employed, and 92.38% had a nuclear family.

In the present study, it was found that while the severity (*p* = 0.001) and benefit (*p* = 0.001) subscale scores of the pregnant women who were primary school graduates were lower (*p* = 0.001), the barrier subscale scores were higher (*p* = 0.001). Moreover, pregnant women with higher education levels had more positive opinions about getting the COVID-19 vaccine during pregnancy (*p* < 0.05).

Severity (*p* = 0.001) and benefit (*p* = 0.001) subscale scores were significantly higher, and barrier (*p* = 0.001) subscale scores were lower in pregnant women in the first trimester compared to those in the second and third trimesters.

**Table 1 -:** Distribution of the pregnant women according to their socio-demographic and obstetric characteristics (n = 407). Nicosia, Cyprus, 2022

**Socio-demographic and obstetric characteristics**	**n** *	**%** ^†^
**Age group** ( **mean 27.61±4.73 years)**		
24 years and younger	85	20.88
25-28 years	172	42.26
29 years and older	150	36.86
**Educational background**		
Primary School	27	6.63
Secondary School	55	13.51
High School	75	18.43
University	215	52.83
Postgraduate	35	8.60
**Income level**		
Income less than the expense	68	16.71
Income equal to the expense	307	75.43
Income higher than the expense	32	7.86
**Employment status**		
Yes	292	71.74
No	115	28.26
**Type of employment**		
Full-time	229	56.27
Part-time	178	43.73
**Type of family**		
Nuclear family	376	92.38
Extended family	31	7.62
**Time married**		
0-3 years	216	53.07
4-10 years	136	33.42
11 years or more	55	13.51
**Gestational age**		
1 ^st^ trimester	111	27.27
2 ^nd^ trimester	163	40.05
3 ^rd^ trimester	133	32.68
**Pregnancy**		
First	254	62.41
Second	118	28.99
Third or more	35	8.60
**Number of living children**		
None	257	63.14
One	115	28.26
Two or more	35	8.60

^*^n = Number of subjects; ^†^% = Percentage

It was determined that 89.43% of the pregnant women included in the study were not vaccinated against tetanus and 100.0% were not vaccinated against influenza. More than half, 51.84% have had the COVID-19 vaccine before pregnancy, 36.12% had positive opinions about getting the COVID-19 vaccine during pregnancy, and 63.88% had negative opinions about being vaccinated against COVID-19 during pregnancy. A fraction of 61.90% of the pregnant women who had a positive opinion believed that the vaccine protects against the COVID-19 virus during pregnancy and 75% of those who had a negative opinion believed that the vaccine would harm the fetus. It was observed that 83.29% of the pregnant women received information about the COVID-19 vaccine during pregnancy and 60.69% stated that they had sufficient knowledge about the COVID-19 vaccine. Most of them, 86.73% of the pregnant women, stated that they obtained information about COVID-19 from a doctor, 45.45% from social media, 55.04% from specialists through TV or Internet, and 28.99% from nurses (Table 2).

**Table 2 -:** Distribution of pregnant women according to their vaccination status and some COVID-19-related characteristics (n = 407). Nicosia, Cyprus, 2022

Vaccination status and COVID-19-related characteristics	**n** *	**%** ^†^
**Being vaccinated against tetanus**		
Yes	43	10.57
No	364	89.43
**Being vaccinated against influenza**		
No	407	100.00
**Paying attention to hand hygiene**		
Yes	407	100.00
**Paying attention to social distancing**		
Yes	407	100.00
**Paying attention to using a facial mask**		
Yes	407	100.00
**Having contracted COVID-19**		
Yes	78	19.16
No	329	80.84
**Having contracted COVID-19 during pregnancy**		
Yes	52	12.78
No	355	87.22
**Personal status regarding the COVID-19 vaccine**		
I received a COVID-19 vaccine before pregnancy	211	51.84
I received a COVID -19 vaccine both before and during pregnancy	32	7.86
I have not been vaccinated yet. I plan to be vaccinated during my pregnancy	41	10.07
I plan to get vaccinated after pregnancy	32	7.86
I plan to get vaccinated after my breastfeeding period	40	9.83
I do not plan to receive a COVID -19 vaccine	51	12.53
**View on receiving a COVID-19 vaccine during pregnancy**		
Negative	260	63.88
Positive	147	36.12
**Reason for a positive opinion (n=147)**		
I believe the vaccine protects me from COVID-19 during pregnancy	91	61.90
I believe the vaccine protects my infant from COVID -19 during pregnancy	56	38.10
**Reason for a negative opinion (n=260)**		
I believe the vaccine would harm my infant	195	75.00
I believe the vaccine poses risk to my infant’s life	123	47.31
I believe the vaccine poses risk to my infant’s mental development	107	41.15
**Obtained information about COVID-19 vaccine during pregnancy**		
Yes	339	83.29
No	68	16.71
**Did you have sufficient knowledge about the COVID-19 vaccine**		
Yes	247	60.69
No	160	39.31
**What information sources on COVID-19 did you have**		
Doctor	353	86.73
Social media	185	45.45
Specialists through TV or Internet	224	55.04
Nurse	118	28.99
Family and friends	63	15.48

^*^n = Number of respondants; ^†^% = Percentage

The mean severity and benefit subscale scores of women who were vaccinated before pregnancy, who were vaccinated both before and during pregnancy, those who were not yet vaccinated, and those who plan to be vaccinated during pregnancy were found to be significantly higher, and barrier subscale mean scores were found to be significantly higher (*p* = 0.001). The mean severity and benefit subscale scores of those who had a positive opinion about receiving the COVID-19 vaccine during pregnancy were found to be higher than those who had a negative opinion (*p* = 0.001). It was determined that those who received information about the COVID-19 vaccine during pregnancy had higher mean scores on the severity subscale (*p* = 0.018). Those who learned about the COVID-19 vaccine during pregnancy and believed that they had sufficient information were found to have higher mean scores of severity and proficiency subscales, and lower mean scores of barrier subscales (*p* < 0.05) (Table 3).

**Table 3 -:** Comparison of the severity, barrier, and benefit subscale mean scores of the COVID-19 vaccine knowledge and attitude scale according to their vaccination status and some COVID-19-related characteristics (n = 407). Nicosia, Cyprus, 2022

**Vaccination status and COVID-19-related characteristics**	**Severity**			**Barrier**			**Benefit**		
	x̄*	SD ^†^	p ^‡^	x̄*	SD ^†^	p ^‡^	x̄*	SD ^†^	p ^‡^
Having COVID-19 infection
Yes	16.00	4.20	0.062	22.36	3.84	0.886	12.44	3.83	0.019
No	18.01	4.66		22.92	5.53		11.93	3.83	
**Having COVID-19 during pregnancy**									
Yes	15.00	4.85	0.003	23.54	4.24	0.029	11.15	4.13	0.224
No	18.01	4.48		22.71	5.38		12.16	3.77	
**Personal status regarding COVID-19 vaccine**									
Being vaccinated before pregnancy	18.97	3.95	0.001	22.42	4.43	0.001	13.00	3.21	0.001
Planning to be vaccinated both before and during pregnancy	20.25	2.95		18.88	5.41		15.88	2.46	
Planning to be vaccinated during pregnancy, since she has not yet been vaccinated	19.83	4.54		19.15	2.02		13.61	1.51	
Planning to be vaccinated after pregnancy	17.50	1.97		22.00	3.93		12.50	1.97	
Planning to be vaccinated after breastfeeding period	13.50	4.56		23.00	5.06		9.50	3.54	
Never thought of receiving a COVID-19 vaccine	11.96	2.47		30.25	3.45		6.00	0.00	
**Opinion on receiving a COVID-19 vaccine during pregnancy**									
Negative	16.15	4.47	0.001	24.32	5.62	0.001	10.77	4.10	0.001
Positive	20.22	3.70		20.16	3.07		14.26	1.74	
**Obtained information about COVID-19 vaccine during pregnancy**									
Yes	17.87	4.93	0.018	22.65	4.94	0.500	12.14	3.81	0.443
No	16.38	2.36		23.62	6.59		11.50	3.92	
**Consider that has enough information about COVID-19 vaccine**									
Yes	18.36	4.77	0.001	22.17	5.30	0.031	12.30	4.05	0.031
No	16.48	4.18		23.81	5.03		11.61	3.42	

^*^x- = Mean; ^†^SD = Standard Deviation; ^‡^ *p* < 0.05, estimated by means of the Wald test

## Discussion

This study’s results reveal that although women have positive attitudes toward vaccinations while not pregnant, this is not the case during pregnancy. It is also observed that the participants do not have a positive attitude towards tetanus and influenza vaccines during pregnancy and prefer to not be vaccinated during pregnancy. The fact that the rate of routine tetanus vaccination during pregnancy is so low suggests that the issue of vaccination during pregnancy may have been overlooked. Different studies have also reported that the seasonal influenza vaccine is not common among pregnant women and is mostly not accepted by pregnant women^27-30^. Furthermore, it was found that 63.88% of the pregnant women had a negative opinion about being vaccinated against COVID-19 during pregnancy, 75% of these pregnant women believed that the vaccine would harm their fetus, and 47.31% thought that the vaccine imposes a risk to their infant’s life. Studies found that pregnant women consider that vaccines could harm themselves or the fetus, cause infertility, and the risk of contracting COVID-19 was low during pregnancy^(7,31)^. Particularly, perceptions about the effects of the vaccine on the fetus can negatively affect the attitude of pregnant women to the vaccine. In a study, unlike our study, the vaccination rate during pregnancy was found to be high, and it was concluded that despite the reservations about the COVID-19 vaccine, a determined and positive attitude towards vaccines in general increased the acceptance of the COVID-19 vaccine^10^.

The severity and benefit subscale scores of pregnant women in the first trimester were found to be higher. In the literature, it has been shown that different results were obtained when vaccine acceptance is evaluated according to gestational age. A study in Turkey found that vaccine acceptance was higher in the first trimester^24^. A study conducted in China indicated that pregnant women in the second and third trimesters were more willing to receive the COVID-19 vaccine than those in the first trimester^32^.

It has been observed that pregnant women with a high level of education had a positive perspective of the COVID-19 vaccine, the severity and benefit subscale scores of the COVID-19 vaccine were higher, and the barrier subscale scores were low. The studies conducted with both pregnant women and the general population have shown that high education level positively affects vaccine acceptance^(25,30,33)^. In addition, the severity and benefit subscale scores of the pregnant women who stated that they had sufficient information about COVID-19 were found to be higher, and the barrier subscale scores were lower. This shows that educating pregnant women about COVID-19 positively affected the approach to vaccination. Different studies support the result of the present study^(10,25,34)^. The results in the literature also show that pregnant women must have adequate information about the efficacy and safety of the COVID-19 vaccine^35-36^.

It was found that pregnant women attached great importance to paying attention to hand hygiene, use of facial masks, and social distancing, from preventive measures other than vaccination. Likewise, a study found that pregnant women paid attention to the use of masks, hand washing, and social distancing at a high rate^25^. These results showed that pregnant women pay attention to preventive methods for COVID-19.

While 39.31% of the pregnant women stated that they did not have enough knowledge about the COVID-19 vaccine, and they obtained information mostly from doctors (86.73%), which was followed by specialists through the TV and Internet (55.04%). In a study, the main source of information was the media^24^. Especially during the lockdown periods, the Internet was widely used as a media information source. However, these sources can also lead to misinformation. As misinformation continues to spread, recommendations from healthcare professionals as a reliable source of information for pregnant women are crucial^37^. A randomized controlled study evaluating the attitudes of pregnant women towards vaccination by sending text messages about the COVID-19 vaccine was found to have a positive effect on the attitudes of pregnant women towards vaccination by sending information messages by healthcare professionals^38^. The rate of pregnant women who stated that they received information about COVID-19 vaccine from nurses is approximately 30%. Nurses work in health institutions where preventive health services are provided and, in addition, they have health education duties. Therefore, the proportion of pregnant women who received information about COVID-19 vaccine from nurses is thought-provoking.

This study is important in terms of showing that negative attitudes towards vaccines during pregnancy may be a similar approach in terms of acceptance of new vaccines. Education and awareness-raising studies on vaccine acceptance and vaccine attitudes during pregnancy should be conducted, especially outside of epidemic periods, and the continuity of these studies should be ensured. Furthermore, our study reveals that nurses should use their role as educators to reach more women about vaccines during pregnancy.

The language of the study is Turkish. Therefore, the results of the study cannot be generalized to the entire island. Since this study was conducted online and the survey was shared via social media, pregnant women with internet connection and using social media were included in this study. Another limitation is that the responses to the questions depend on the individuals’ subjectivity, since the data was collected online.

## Conclusion

In the present study, it was determined that pregnant women had a negative attitude toward vaccination during pregnancy and this negative attitude persisted against COVID-19 vaccines. The most prominent reason for the negative attitude toward COVID-19 vaccines was the concern that it may adversely affect the pregnancy and harm the fetus. The higher the education level of pregnant women, the more positive attitudes they had about being vaccinated against COVID-19 during pregnancy. Moreover, getting enough information about the COVID-19 vaccine affected the attitudes of pregnant women about being vaccinated. In line with these results, it is important to provide training to pregnant women to protect them in the disadvantaged group and to ensure the sustainability of COVID-19 vaccines. Providing information about COVID-19 vaccine and its effects alone will not suffice to prevent new outbreaks. It was found that the vast majority of those who have a negative attitude towards COVID-19 vaccine believe that the vaccine will harm their pregnancy. In addition, the fact that other vaccines are at very low rates during pregnancy reveals that they have a negative attitude against being vaccinated during pregnancy. Therefore, information about the importance of being vaccinated during pregnancy to protect the fetus’ and the mother’s health should not be ignored. It is also recommended that qualitative studies including in-depth interviews be conducted to determine women’s concerns to vaccination during pregnancy.
