# Supplementary material for: Knowledge and attitudes of pregnant women about COVID-19 vaccination
Source: Rev Lat Am Enfermagem. 2025 May 2;33:e4521. doi: 10.1590/1518-8345.7331.4521 (PMC12052313; doi:10.1590/1518-8345.7331.4521)
Supplement: Translation

# Translation: Conhecimento e atitudes de gestantes sobre a vacinação contra a COVID-19

**DOI:** 10.1590/1518-8345.7331.4522

**Authors:** Serap Tekbaş

**Affiliations:** 1 Izmir Tinaztepe University, Faculty of Health Sciences, İzmir, Turquia

**Keywords:** Vacina, COVID-19, Mulheres, Gravidez, Conhecimento, Atitude

## Abstract

avaliar o conhecimento e as atitudes de gestantes em relação à vacinação contra a COVID-19 e os fatores que os afetam.

este é um estudo transversal e analítico com 407 gestantes. A Escala de Conhecimento e Atitude em relação à Vacina contra a COVID-19 foi usada para avaliar o conhecimento e as atitudes das gestantes em relação à vacinação contra a COVID-19. Os testes U de Mann-Whitney e H de Kruskal-Wallis foram usados para análise estatística.

verificou-se que 63,88% da amostra tinha uma opinião negativa sobre a vacinação contra a COVID-19 durante a gravidez. Setenta e cinco por cento das gestantes acreditavam que a vacina poderia prejudicar o feto. Os escores médios da subescala de gravidade (*p* = 0,001) e benefício (*p* = 0,001) da vacina foram significativamente mais altos em gestantes com maior nível de escolaridade e no primeiro trimestre da gravidez (*p* = 0,001). Verificou-se que as gestantes que afirmaram ter informação suficiente sobre a COVID-19 apresentaram escores médios da subescala de gravidade (*p* = 0,001) e benefício (*p* = 0,031) significativamente mais altos e tiveram uma atitude mais positiva em relação à vacinação contra a COVID-19 durante a gravidez.

a maioria das mulheres grávidas teve atitudes negativas devido à preocupação de que a vacina contra a COVID-19 pudesse prejudicar o feto. Os profissionais de saúde devem educar as gestantes sobre a importância da vacinação durante a gravidez, em benefício da saúde materna e infantil durante o acompanhamento da gestação.

## Destaques

**(1)** O estudo avaliou a aceitação da vacina e a atitude em relação à vacina contra a COVID-19 durante a gravidez. **(2)** Mulheres gestantes participaram do estudo. **(3)** Foram detectadas atitudes negativas em relação às vacinas durante a gravidez. **(4)** Os resultados do estudo podem se tornar um instrumento para informar as instituições de saúde sobre a aceitação da vacina.

## Introdução

A COVID-19, causada pelo coronavírus 2 da síndrome respiratória aguda grave (SARS-CoV-2), tem causado morbidade e mortalidade maciças em todo o mundo^1^. Desde 2019, quando os primeiros casos de coronavírus foram detectados, até julho de 2024, mais de 775 milhões de casos confirmados e mais de 7 milhões de mortes foram registrados em todo o mundo. Ademais, aproximadamente 14 bilhões de doses da vacina contra a COVID-19 foram administradas^2^.

Devido às mudanças fisiológicas e imunológicas que ocorrem durante a gravidez, as mulheres grávidas se tornam mais suscetíveis a infecções do trato respiratório, o que as coloca em alto risco de graves consequências no caso de contraírem COVID-19^3^.

Estudos relataram que a infecção por COVID-19 piora o curso clínico em mulheres grávidas em comparação com mulheres não grávidas da mesma idade^4^. Os Centros de Controle e Prevenção de Doenças (CDC) dos EUA relatam que mulheres grávidas têm três vezes mais chances de serem admitidas em unidades de terapia intensiva ou de precisarem de intubação e 1,5 vezes mais chances de morrerem de COVID-19 do que mulheres não grávidas^5^.

Os efeitos adversos da COVID-19 também foram encontrados em termos da saúde e desenvolvimento fetal. Em uma metanálise, constatou-se que mulheres grávidas infectadas pelo SARS-CoV-2 tinham maior risco de produzir natimorto e parto prematuro do que mulheres grávidas não infectadas^6-7^. Além disso, a infecção pelo SARS-CoV-2 pode desencadear uma tempestade de citocinas, que pode levar a uma resposta inflamatória no feto e a danos na placenta com subsequente restrição do crescimento fetal, trabalho de parto prematuro e aborto espontâneo^8^.

Muitos fatores afetam as atitudes dos indivíduos em relação à vacinação. Estes fatores incluem o tipo de vacina, a localização geográfica, a cultura e o status socioeconômico. As pessoas também podem ter reservas quanto à aceitação de novas vacinas. A hesitação vacinal pode aumentar durante a gravidez devido à preocupação com seus efeitos sobre o feto^9-10^.

Uma das abordagens mais eficazes contra a COVID-19, que provoca a síndrome respiratória aguda grave, é a vacinação^10-11^. Como nenhuma das vacinas contra a COVID-19 contém vírus vivos ou adjuvantes que possam prejudicar o feto, inúmeros estudos, o *American College of Obstetricians and Gynecologists* ^(5,12-14)^ e a *Society for Maternal-Fetal Medicine* recomendam a vacinação a mulheres grávidas e lactantes^15-16^. Embora o *American College of Obstetricians and Gynecologists* recomende a vacina contra a COVID-19 para todas as gestantes, a decisão deve ser deixada a cargo da mulher, depois que os fatores de risco individuais forem cuidadosamente avaliados^5^. A literatura enfatiza que as reações relacionadas à vacina em gestantes e lactantes são semelhantes às da população em geral. Além disso, os resultados da pesquisa relatam que as gestantes vacinadas têm menos probabilidade de contrair a COVID-19 e que caso a contraiam, a doença tem um curso mais brando nas gestantes e em bebês nascidos de mães vacinadas^17^. As mulheres grávidas geralmente têm menos disposição e maior preocupação com a vacinação contra doenças evitáveis por vacina (por exemplo, influenza e tétano) do que a população em geral^10^. Estudos indicam que mulheres que têm uma atitude negativa em relação às vacinas durante a gravidez também têm hesitação em relação à vacina contra a COVID-19^4^. Esta situação mostra que o desenvolvimento de uma atitude positiva em relação à vacinação durante a gravidez pode reduzir a hesitação vacinal contra a COVID-19 e, potencialmente, a novas vacinas^18-20^.

Considerando o aumento da morbidade associada à COVID-19 na gravidez, é importante determinar o conhecimento e as atitudes das gestantes em relação à vacinação contra a COVID-19 durante a gravidez, a fim de proteger a gestante e o feto da COVID-19 e reduzir seus efeitos adversos na gravidez e no feto^21-22^. Por estes motivos, este estudo foi planejado para avaliar o conhecimento e as atitudes de gestantes em relação à vacina contra a COVID-19 e os fatores que afetam seu conhecimento e suas atitudes.

## Métodos

### Tipo de estudo

Esta pesquisa é um estudo descritivo, transversal e analítico. Ela se baseia nas diretrizes do *Strengthening the Reporting of Observational Studies in Epidemiology* (STROBE).

### Local e população do estudo

O estudo foi realizado de janeiro a abril de 2022 no norte do Chipre, que é uma ilha de 9.251 km^2^ no Mediterrâneo, onde o turismo é generalizado e há muitos alunos matriculados nas 22 universidades. Por estes motivos, a população de estudantes, tanto acadêmicos quanto profissionais, é bastante alta. Além disso, o Norte do Chipre é uma ilha com uma taxa de natalidade muito baixa e, de acordo com dados oficiais, o número total de nascimentos no país em 2012 foi de 3.614^24^. Os partos de mulheres que têm status de imigrantes para fins de educação e trabalho também estão incluídos neste número.

Mulheres grávidas com mais de 18 anos de idade que participaram voluntariamente do estudo foram incluídas no estudo, independentemente de sua idade gestacional. Os dados foram coletados *online* para evitar o risco de infecção devido à pandemia. As pesquisas foram elaboradas pelos pesquisadores usando o *Google Forms*. O *link* para o Formulário *Google* criado foi compartilhado pelo pesquisador nas plataformas de mídia social (*WhatsApp*, *Messenger*, *Facebook* e *Instagram*). A primeira página do questionário *online* incluía informação sobre a finalidade e o conteúdo do estudo e o formulário de consentimento informado para participação no estudo. O formulário foi criado de forma que aqueles que concordassem em participar continuassem s responder ao questionário.

### Seleção da amostra

O número de gestantes a serem incluídas na amostra foi determinado pelo método de análise de poder. As estatísticas da taxa de natalidade do país foram usadas como referência para a análise do poder estatístico^23^. O número de gestantes necessárias para o estudo foi de 365 com α=0,05, 1-β=0,80 e taxa de erro de 0,20. Das mulheres convidadas para o estudo, 407 aceitaram participar e constituíram o grupo de amostra do estudo. Após a conclusão do estudo, foi realizada uma análise de poder *post-hoc* para determinar a adequação do tamanho da amostra. De acordo com a análise *post-hoc*, foi determinado que a amostra total era suficiente com um tamanho de efeito de 0,92, poder de 99% e margem de erro de 0,05%.

### Critérios de inclusão

Mulheres grávidas com idade igual ou superior a 18 anos, sem barreiras de comunicação (que não falam turco, problemas de saúde mental etc.), alfabetizadas e com alfabetização digital básica foram incluídas no estudo.

### Critérios de exclusão

Mulheres grávidas com quaisquer fatores de risco durante a gravidez (por exemplo, pré-eclâmpsia, retardo de crescimento intrauterino, ruptura prematura de membranas, diabetes gestacional etc.) e mulheres grávidas com quaisquer problemas diagnosticados relacionados à saúde do feto (por exemplo, anomalia fetal e retardo de crescimento intrauterino) foram excluídas.

### Ferramentas de coleta de dados

Os primeiros 14 itens do formulário com 27 perguntas preparado pelo pesquisador após a revisão da literatura foram classificados para determinar as características sociodemográficas dos participantes^(20,24)^. Treze perguntas incluíram o status de vacinação das participantes, suas opiniões sobre a vacinação contra a COVID-19 durante a gravidez e as fontes de informação sobre a vacina^(1,7,24-25)^.

#### Escala de Conhecimento e Atitude em relação à Vacina contra a COVID-19

A escala, desenvolvida em 2021 para avaliar o conhecimento e a atitude dos indivíduos em relação à vacina contra a COVID-19, é do tipo Likert de cinco pontos e consiste em 16 perguntas^26^. A escala não tem ponto de corte. A alta pontuação média obtida em cada subescala indica que as percepções em relação à gravidade, ao obstáculo e ao benefício da subescala são altas. A escala consiste em um total de três subescalas: “Percepção da gravidade da doença COVID-19” (5 itens), “Percepção dos obstáculos da vacina” (7 itens) e “Percepção do benefício da vacina” (4 itens). Na pontuação da escala, cada subescala é pontuada por si só. Enquanto as pontuações altas nas subescalas de gravidade e benefício indicam uma atitude positiva em relação à COVID-19, uma pontuação alta na subescala de barreira indica uma atitude negativa em relação à COVID-19. O coeficiente alfa de Cronbach da escala usada em nosso estudo foi calculado em 0,83.

### Análise dos dados

No estudo, as características sociodemográficas e o status de vacinação das gestantes e sua distribuição de acordo com algumas características relacionadas à COVID-19 foram determinados pela análise de frequência. As estatísticas descritivas referentes aos escores da Escala de Conhecimento e Atitude em relação à Vacina contra a COVID-19 das gestantes foram fornecidas e a conformidade com a distribuição normal foi examinada com o teste de Kolmogorov-Smirnov. Testes de hipóteses não paramétricos, como os testes U de Mann-Whitney e H de Kruskal-Wallis, foram usados no estudo porque os escores da Escala de Conhecimento e Atitude em relação à vacina contra a COVID-19 das gestantes não apresentaram uma distribuição normal.

### Aspectos éticos

Este estudo foi aprovado pelo Comitê de Revisão Institucional da Universidade (nº 2021/98-1444). Antes de iniciar a pesquisa, obteve-se o consentimento de todos os participantes e a confidencialidade das informações pessoais foi assegurada. O estudo foi conduzido de acordo com os princípios éticos da Declaração de Helsinki.

## Resultados

A Tabela 1 apresenta as características sociodemográficas do estudo realizado com a participação de 407 gestantes. A média de idade das gestantes participantes do estudo foi de 27,61±4,73 anos. Foi determinado que 27,27% das participantes estavam no primeiro trimestre, 40,05% no segundo trimestre e 32,68% no terceiro trimestre. Uma fração de 62,41% estava em sua primeira gravidez, 28,99% estavam em sua segunda gravidez e 28,26% já tinham um filho.

Determinou-se que 13,51% das gestantes tinham ensino secundário completo, 18,43% tinham ensino médio completo e 52,83% tinham ensino superior completo. Cerca de dois terços, 75,43%, tinham uma renda equivalente às despesas, 71,74% estavam empregadas e 92,38% tinham uma família nuclear.

No presente estudo, verificou-se que, embora os escores das subescalas de gravidade (*p* = 0,001) e benefício (*p* = 0,001) das gestantes que concluíram o ensino fundamental tenham sido menores (*p* = 0,001), os escores da subescala de barreira foram maiores (*p* = 0,001). Além disso, as gestantes com nível de escolaridade mais alto tinham opiniões mais positivas sobre a vacina contra a COVID-19 durante a gravidez (*p* < 0,05). As pontuações da subescala de gravidade (*p* = 0,001) e benefício (*p* = 0.001) foram significativamente mais altas, e as pontuações da subescala de barreira (*p* = 0,001) foram mais baixas em gestantes no primeiro trimestre em comparação com as do segundo e terceiro trimestres.

**Tabela 1 -:** Distribuição das gestantes de acordo com suas características sociodemográficas e obstétricas (n = 407). Nicósia, Chipre, 2022

**Características sociodemográficas e obstétricas**	**n** *	**%** ^†^
**Faixa etária (média 27,61±4,73 anos)**		
24 anos ou menos	85	20,88
25-28 anos	172	42,26
29 anos ou mais	150	36,86
**Formação educacional**		
Ensino fundamental	27	6,63
Ensino secundário	55	13,51
Ensino médio	75	18,43
Graduação	215	52,83
Pós-graduação	35	8,60
**Nível de renda**		
Renda menor que a despesa	68	16,71
Renda igual à despesa	307	75,43
Renda maior que a despesa	32	7,86
**Empregado**		
Sim	292	71,74
Não	115	28,26
**Tipo de emprego**		
Tempo integral	229	56,27
Tempo parcial	178	43,73
**Tipo de família**		
Família nuclear	376	92,38
Família estendida	31	7,62
**Tempo de casamento**		
0-3 anos	216	53,07
4-10 anos	136	33,42
11 anos ou mais	55	13,51
**Idade gestacional**		
1º trimestre	111	27,27
2º trimestre	163	40,05
3º trimestre	133	32,68
**Gravidez**		
Primeira	254	62,41
Segunda	118	28,99
Terceira ou mais	35	8,60
**Número de filhos vivos**		
Nenhum	257	63,14
Um	115	28,26
Dois ou mais	35	8,60

^*^n = Número de casos; ^†^% = Percentagem

Foi determinado que 89,43% das gestantes incluídas no estudo não foram vacinadas contra o tétano e 100% não foram vacinadas contra a influenza (gripe). Mais da metade, 51,84%, tomou a vacina contra a COVID-19 antes da gravidez, 36,12% tiveram opiniões positivas sobre a vacinação contra a COVID-19 durante a gravidez, enquanto 63,88% tiveram opiniões negativas. Uma fração de 61,90% das gestantes que tinham opinião positiva acreditava que a vacina protege contra o vírus da COVID-19 durante a gravidez e 75% das que tinham uma opinião negativa, acreditavam que a vacina prejudicaria o feto. Foi observado que 83,29% das gestantes receberam informação sobre a vacina contra a COVID-19 durante a gravidez e 60,69% afirmaram ter conhecimento suficiente sobre a vacina contra a COVID-19. A maioria, 86,73% das gestantes, declarou que obteve informação sobre a vacina contra a COVID-19 com um médico, 45,45% nas mídias sociais, 55,04% com especialistas pela TV ou Internet e 28,99% com enfermeiros (Tabela 2).

**Tabela 2 -:** Distribuição das gestantes de acordo com seu status de vacinação e algumas características relacionadas à COVID-19 (n = 407). Nicósia, Chipre, 2022

Status de vacinação e características relacionadas à COVID-19	**n** *	**%** ^†^
**Está vacinada contra o tétano**		
Sim	43	10,57
Não	364	89,43
**Está vacinada contra a influenza**		
Não	407	100,00
**Presta atenção à higiene das mãos**		
Sim	407	100,00
**Presta atenção ao distanciamento social**		
Sim	407	100,00
**Presta atenção ao uso de máscara facial**		
Sim	407	100,00
**Contraiu COVID-19**		
Sim	78	19,16
Não	329	80,84
**Contraiu COVID-19 durante a gravidez**		
Sim	52	12,78
Não	355	87,22
**Status pessoal referente à vacina contra a COVID-19**		
Recebi a vacina contra a COVID-19 antes da gravidez	211	51,84
Recebi a vacina contra a COVID-19 antes e durante a gravidez	32	7,86
Ainda não fui vacinada. Pretendo ser vacinada durante a gravidez	41	10,07
Pretendo ser vacinada após a gravidez	32	7,86
Pretendo ser vacinado após o período de amamentação	40	9,83
Não pretendo receber a vacina contra a COVID-19	51	12,53
**Opinião sobre receber a vacina contra a COVID-19 durante a gravidez**		
Negativa	260	63,88
Positiva	147	36.12
**Motivo para opinião positiva (n=147)**		
Acredito que a vacina me protegerá contra a COVID-19 durante a gravidez	91	61,90
Acredito que a vacina protegerá meu bebê da COVID-19 durante a gravidez	56	38,10
**Motivo para opinião negativa (n=260)**		
Acredito que a vacina prejudicaria meu bebê	195	75,00
Acredito que a vacina representa um risco para a vida do meu bebê	123	47,31
Acredito que a vacina representa um risco para o desenvolvimento mental do meu bebê	107	41,15
**Obteve informação sobre a vacina contra a COVID-19 durante a gravidez**		
Sim	339	83,29
Não	68	16,71
**Você tinha conhecimento suficiente sobre a vacina contra a COVID-19**		
Sim	247	60,69
Não	160	39,31
**Quais fontes de informação sobre a COVID-19 você consultava**		
Médico	353	86,73
Redes sociais	185	45,45
Especialistas por meio de TV/Internet	224	55,04
Enfermeiros	118	28,99
Familiares e amigos	63	15,48

^*^n = Número de respondentes; ^†^% = Percentagem

As pontuações médias das subescalas de gravidade e benefício das mulheres que foram vacinadas antes da gravidez, que foram vacinadas antes e durante a gravidez, das que ainda não foram vacinadas e das que planejam ser vacinadas durante a gravidez, foram significativamente mais altas, e as pontuações médias das subescalas de barreira foram significativamente mais altas (*p* = 0,001). As pontuações médias da subescala de gravidade e benefício das que tinham uma opinião positiva sobre receber a vacina contra a COVID-19 durante a gravidez foram maiores do que as das que tinham uma opinião negativa (*p* = 0,001). Foi determinado que aquelas que receberam informação sobre a vacina contra a COVID-19 durante a gravidez tiveram escores médios mais altos na subescala de gravidade (*p* = 0,018). Verificou-se que aquelas que foram instruídas sobre a vacina contra a COVID-19 durante a gravidez e acreditavam que tinham informação suficiente apresentavam pontuações médias mais altas nas subescalas de gravidade e proficiência e pontuações médias mais baixas nas subescalas de barreira (*p* < 0,05) (Tabela 3).

**Tabela 3 -:** Comparação das pontuações médias das subescalas de gravidade, barreira e benefício da escala de conhecimento e atitude em relação à vacina contra a COVID-19 de acordo com o status de vacinação e algumas características relacionadas à COVID-19 (n = 407). Nicósia, Chipre, 2022

**Status de vacinação e características relacionadas à COVID-19**	**Gravidade**			**Barreira**			**Benefício**		
	**x̄** *	**DP** ^†^	***p*** ^‡^	**x̄** *	**DP** ^†^	***p*** ^‡^	x̄*	**DP** ^†^	***p*** ^‡^
**Contraiu a infecção pela COVID-19**									
Sim	16,00	4,20	0,062	22,36	3,84	0,886	12,44	383	0,019
Não	18,01	4,66		22,92	5,53		11,93	3,83	
**Contraiu a infecção pela COVID-19 durante a gestação**									
Sim	15,00	4,85	0,003	23,54	4,24	0,029	11,15	4,13	0,224
Não	18,01	4,48		22,71	5,38		12,16	3,77	
**Status pessoal com relação à vacina contra a COVID-19**									
Já foi vacinada antes da gravidez	18,97	3,95	0,001	22,42	4,43	0,001	13,00	3,21	0,001
Planeja ser vacinada antes e durante a gravidez	20,25	2,95		18,88	5,41		15,88	2,46	
Planejando ser vacinada durante a gravidez, já que ainda não foi vacinada	19,83	4,54		19,15	2,02		13,61	1,51	
Planejando ser vacinada após a gravidez	17,50	1,97		22,00	3,93		12,50	1,97	
Planejando ser vacinada após o período de amamentação	13,50	4,56		23,00	5,06		9,50	3,54	
Nunca pensou em tomar a vacina contra a COVID-19	11,96	2,47		30,25	3,45		6,00	0,00	
**Opinião sobre receber a vacina contra a COVID-19 durante a gravidez**									
Negativa	16,15	4,47	0,001	24,32	5,62	0,001	10,77	4,10	0,001
Positiva	20,22	3,70		20,16	3,07		14,26	1,74	
**Obteve informação sobre a vacina contra a COVID-19 durante a gravidez**									
Sim	17,87	4,93	0,018	22,65	4,94	0,500	12,14	3,81	0,443
Não	16,38	2,36		23,62	6,59		11,50	3,92	
**Considera que tem informação suficiente sobre a vacina contra a COVID-19**									
Sim	18,36	4,77	0,001	22,17	5,30	0,031	12,30	4,05	0,031
Não	16,48	4,18		23,81	5,03		11,61	3,42	

^*^x- = Média; ^†^DP = Desvio-Padrão; ^‡^ *p* < 0,05, estimado por meio do teste Wald

## Discussão

Os resultados deste estudo revelam que, embora as mulheres tenham atitudes positivas em relação às vacinas quando não estão grávidas, este não é o caso durante a gravidez. O fato de a taxa de vacinação de rotina contra o tétano durante a gravidez ser tão baixa sugere que a questão da vacinação durante a gravidez pode ter sido negligenciada. Diferentes estudos também relataram que a vacina contra a gripe sazonal não é comum entre as gestantes e, na maioria das vezes, não é aceita por elas^27-30^. Além disso, verificou-se que 63,88% das gestantes tinham uma opinião negativa sobre a vacinação contra a COVID-19 durante a gravidez, sendo que 75% destas gestantes acreditavam que a vacina poderia prejudicar o feto e 47,31% achavam que a vacina poderia impor um risco à vida do bebê. Estudos constataram que as gestantes consideram que as vacinas poderiam prejudicar a si mesmas ou ao feto, causar infertilidade e que o risco de contrair a COVID-19 era baixo durante a gravidez^(7,31)^. Particularmente, as percepções sobre os efeitos da vacina no feto podem afetar negativamente a atitude das gestantes em relação à vacina. Em um estudo, ao contrário do nosso, a taxa de vacinação durante a gravidez foi alta, e concluiu-se que, apesar das reservas sobre a vacina contra a COVID-19, uma atitude determinada e positiva em relação às vacinas em geral aumentou a aceitação da vacina contra a COVID-19^10^.

As pontuações das subescalas de gravidade e benefício de mulheres grávidas no primeiro trimestre foram consideradas maiores. Na literatura, foi demonstrado que resultados diferentes foram obtidos quando a aceitação da vacina é avaliada de acordo com a idade gestacional. Um estudo na Turquia descobriu que a aceitação da vacina foi maior no primeiro trimestre^24^. Um estudo conduzido na China indicou que mulheres grávidas no segundo e terceiro trimestres estavam mais dispostas a receber a vacina COVID-19 do que aquelas no primeiro trimestre^32^.

Observou-se que as gestantes com alto nível de escolaridade tinham uma perspectiva positiva da vacina contra a COVID-19, os escores da subescala de gravidade e benefício da vacina contra a COVID-19 eram mais altos e os escores da subescala de barreira eram baixos. Os estudos realizados tanto com gestantes quanto com a população em geral mostraram que o alto nível de escolaridade afeta positivamente a aceitação da vacina^(25,30,33)^. Além disso, os escores da subescala de gravidade e benefício das gestantes que afirmaram ter informação suficiente sobre a COVID-19 foram mais altos, e os escores da subescala de barreira foram mais baixos. Isso mostra que a educação das gestantes sobre a COVID-19 afetou positivamente a abordagem da vacinação. Diferentes estudos apoiam o resultado do presente estudo^(10,25,34)^. Os resultados da literatura também mostram que as gestantes devem ter informação adequada sobre a eficácia e a segurança da vacina contra a COVID-19^35-36^.

Constatou-se que as gestantes atribuíam grande importância à atenção à higiene das mãos, ao uso de máscaras faciais e ao distanciamento social, a partir de medidas preventivas que não a vacinação. Da mesma forma, um estudo constatou que as gestantes prestavam atenção ao uso de máscaras, à lavagem das mãos e ao distanciamento social em uma alta taxa^25^. Estes resultados mostraram que as gestantes prestam atenção aos métodos para prevenir a COVID-19.

Enquanto 39,31% das gestantes afirmaram não ter conhecimento suficiente sobre a vacina contra a COVID-19, declararam ter obtido informação principalmente de médicos (86,73%), seguidos por especialistas por meio da TV e da Internet (55,04%). Em um estudo, a principal fonte de informação foi a mídia^24^. Especialmente durante os períodos de *lockdown*, a Internet foi amplamente usada como fonte de informação da mídia. No entanto, estas fontes também podem levar à desinformação. Como a desinformação continua a se espalhar, as recomendações dos profissionais de saúde como uma fonte confiável de informação para mulheres grávidas são cruciais^37^. Um estudo controlado e randomizado que avaliou as atitudes das gestantes em relação à vacinação por meio do envio de mensagens de texto sobre a vacina contra a COVID-19 demonstrou ter um efeito positivo sobre as atitudes das gestantes em relação à vacinação por meio do envio de mensagens informativas por profissionais de saúde^38^. A taxa de gestantes que declararam ter recebido informação sobre a vacina contra a COVID-19 de enfermeiras é de aproximadamente 30%. A taxa de gestantes que declararam ter recebido informação sobre a vacina contra a COVID-19 de enfermeiras é de aproximadamente 30%. Os enfermeiros trabalham em instituições de saúde onde são prestados serviços de saúde preventiva e, além disso, têm funções de educação em saúde. Portanto, a proporção de gestantes que receberam informação sobre a vacina contra a COVID-19 de enfermeiras é instigante.

Este estudo é importante para mostrar que as atitudes negativas em relação às vacinas durante a gravidez podem ser uma abordagem semelhante em termos de aceitação de novas vacinas. Estudos de educação e conscientização sobre a aceitação e as atitudes em relação às vacinas devem ser conduzidos durante a gravidez, especialmente fora dos períodos epidêmicos, e a continuidade destes estudos deve ser assegurada. Além disso, nosso estudo revela que os enfermeiros devem usar seu papel de educadores para alcançar mais mulheres com informação sobre vacinas durante a gravidez.

O idioma do estudo é o turco, portanto, os resultados do estudo não podem ser generalizados para toda a ilha. Como este estudo foi realizado *online* e a pesquisa foi compartilhada por meio de redes sociais, apenas mulheres grávidas com conexão à Internet e que usam mídias sociais foram incluídas neste estudo. Outra limitação é que a resposta às perguntas depende da subjetividade dos indivíduos, pois os dados foram coletados *online*.

## Conclusão

No presente estudo, foi determinado que as gestantes tinham uma atitude negativa em relação à vacinação durante a gravidez e esta atitude negativa persistiu em relação às vacinas contra a COVID-19. O motivo mais proeminente para a atitude negativa em relação às vacinas contra a COVID-19 foi a preocupação de que elas possam afetar negativamente a gravidez e prejudicar o feto. Quanto maior o nível de escolaridade das gestantes, mais atitudes positivas elas tiveram em relação à vacinação contra a COVID-19 durante a gravidez. Além disso, a obtenção de informação suficiente sobre a vacina contra a COVID-19 afetou as atitudes das gestantes em relação à vacinação. De acordo com estes resultados, é importante oferecer treinamento às gestantes para protegê-las no grupo desfavorecido e garantir a sustentabilidade das vacinas contra a COVID-19. O fornecimento de informação sobre a vacina contra a COVID-19 e seus efeitos, por si só, não será suficiente para evitar novos surtos. Descobriu-se que a grande maioria das pessoas que têm uma atitude negativa em relação à vacina contra a COVID-19 e acredita que a vacina prejudicará sua gravidez. Além disso, o fato de que outras vacinas têm taxas muito baixas durante a gravidez revela que elas têm uma atitude negativa contra a vacinação em geral durante a gravidez. Portanto, a informação sobre a importância da vacinação durante a gravidez para proteger a saúde do feto e da mãe não devem ser ignoradas. Recomenda-se também a realização de estudos qualitativos, incluindo entrevistas aprofundadas, para determinar as preocupações das mulheres sobre a vacinação.

# Translation: Conocimientos y actitudes de gestantes sobre la vacunación contra la COVID-19

**DOI:** 10.1590/1518-8345.7331.4520

**Authors:** Serap Tekbaş

**Affiliations:** 1 Izmir Tinaztepe University, Faculty of Health Sciences, İzmir, Turquía

**Keywords:** Vacuna, COVID-19, Mujeres, Embarazo, Conocimiento, Actitud

## Abstract

evaluar los conocimientos y actitudes de las mujeres embarazadas hacia la vacunación contra la COVID-19 y los factores que los afectan.

se trata de un estudio transversal y analítico de 407 mujeres embarazadas. Se utilizó la Escala de Conocimientos y Actitudes hacia la Vacuna COVID-19 para evaluar los conocimientos y actitudes de las embarazadas hacia la vacunación COVID-19. Para el análisis estadístico se utilizaron las pruebas U de Mann-Whitney y H de Kruskal-Wallis.

el 63,88% de la muestra tenía una opinión negativa de la vacunación con COVID-19 durante el embarazo. El 75% de las embarazadas creía que la vacuna podía dañar al feto. Las puntuaciones medias en las subescalas de gravedad (*p* = 0,001) y beneficio (*p* = 0,001) de la vacuna fueron significativamente más altas en las embarazadas con mayor nivel educativo y en el primer trimestre de embarazo (*p* = 0,001). Se observó que las mujeres embarazadas que afirmaron haber recibido información suficiente sobre la vacuna COVID-19 obtuvieron puntuaciones medias significativamente más altas en las subescalas de gravedad (*p* = 0,001) y beneficio (*p* = 0,031) y tuvieron una actitud más positiva hacia la vacunación contra la COVID-19 durante el embarazo.

la mayoría de las mujeres embarazadas tenían actitudes negativas debido a la preocupación de que la vacuna COVID-19 pudiera dañar al feto. Los profesionales de la salud deberían educar sobre la importancia de la vacunación durante el embarazo en beneficio de la salud materno infantil durante el seguimiento del embarazo.

## Destacados

**(1)** El estudio evaluó la aceptación de la vacuna y las actitudes hacia la vacuna COVID-19 durante el embarazo. **(2)** Las mujeres embarazadas participaron en el estudio. **(3)** Se detectaron actitudes negativas hacia las vacunas durante el embarazo. **(4)** Los resultados del estudio podrían ser una herramienta para informar a las instituciones de salud sobre la aceptación de las vacunas.

## Introducción

COVID-19, causada por el coronavirus 2 del síndrome respiratorio agudo severo (SARS-CoV-2), ha provocado una morbilidad y mortalidad masivas en todo el mundo^(1)^. Desde 2019, cuando se detectaron los primeros casos, hasta julio de 2024, se han registrado más de 775 millones de casos confirmados y más de 7 millones de muertes en todo el mundo. Además, se han administrado aproximadamente 14.000 millones de dosis de vacunas contra la COVID-19^(2)^.

Debido a los cambios fisiológicos e inmunológicos que se producen durante el embarazo, las mujeres embarazadas se vuelven más susceptibles a las infecciones de las vías respiratorias, lo que las expone a un alto riesgo de resultados graves si contraen la COVID-19^(3)^.

Los estudios han informado de que la infección por COVID-19 empeora el curso clínico en las mujeres embarazadas en comparación con las mujeres no embarazadas de la misma edad^(4)^. Los Centros para el Control y la Prevención de Enfermedades (CDC) de los Estados Unidos informan de que las mujeres embarazadas tienen tres veces más probabilidades de ingresar en la unidad de cuidados intensivos o de necesitar intubación y 1,5 veces más probabilidades de morir por COVID-19 que las mujeres no embarazadas^(5)^.

También se han encontrado efectos adversos del COVID-19 en términos de salud y desarrollo fetal. En un estudio de metaanálisis, se observó que las mujeres embarazadas infectadas con el SARS-CoV-2 presentaban un mayor riesgo de mortinatalidad y parto prematuro que las mujeres embarazadas no infectadas^(6-7)^. Además, la infección por SARS-CoV-2 puede desencadenar una tormenta de citocinas, que puede provocar una respuesta inflamatoria en el feto y dañar la placenta, con la consiguiente restricción del crecimiento fetal, potencial parto prematuro y aborto espontaneo^(8)^.

Son muchos los factores que influyen en la actitud de las personas hacia la vacunación. Estos factores incluyen el tipo de vacuna, la región geográfica, la cultura y el estatus socioeconómico. Las personas también pueden tener reservas a la hora de aceptar nuevas vacunas. La reticencia hacia la vacunación puede aumentar durante el embarazo debido a la preocupación por sus efectos en el feto^(9-10)^.

Uno de los enfoques más eficaces contra el COVID-19, causante del síndrome respiratorio agudo grave, es la vacunación^(10-11)^. Puesto que ninguna de las vacunas contra la COVID-19 contiene virus vivos ni adyuvantes que puedan dañar al feto, varios estudios, el *American College of Obstetricians and Gynecologists* ^(5,12-14)^ y la *Society for Maternal-Fetal Medicine* recomiendan la vacunación de las mujeres embarazadas y lactantes^(15-16)^. Aunque el *American College of Obstetricians and Gynecologists* recomienda la vacuna COVID-19 para todas las mujeres embarazadas, se ha afirmado que la decisión debe dejarse en manos de la mujer, después de haber evaluado cuidadosamente los factores de riesgo individuales^(5)^. La literatura destaca que las reacciones relacionadas con la vacuna en mujeres embarazadas y lactantes son similares a las de la población general. Además, los resultados de las investigaciones informan de que las mujeres embarazadas vacunadas tienen menos probabilidades de contraer COVID-19 y que en caso de contraer la enfermedad, esa tiene un curso más leve en las mujeres y los bebés nacidos de madres vacunadas^(17)^. En general, las mujeres embarazadas están menos dispuestas y más preocupadas por la vacunación contra enfermedades prevenibles mediante vacunación (por ejemplo, la influenza y el tétanos) que la población general^(10)^. Se ha determinado que las mujeres que tienen una actitud negativa hacia las vacunas durante el embarazo también dudan hacia la vacuna COVID-19^(4)^. Esta situación demuestra que el desarrollo de una actitud positiva hacia la vacunación durante el embarazo puede reducir la indecisión hacia la vacuna COVID-19 y, potencialmente, hacia nuevas vacunas^(18-20)^.

Teniendo en cuenta el aumento de la morbilidad asociada a la COVID-19 durante el embarazo, es importante determinar los conocimientos y las actitudes de las embarazadas hacia la vacunación contra la COVID-19 durante el embarazo, con el fin de proteger a la embarazada y al feto frente a la COVID-19 y reducir los efectos adversos de la enfermedad sobre el embarazo y el feto^(21-22)^. Por estas razones, este estudio fue diseñado para evaluar los conocimientos y actitudes de las mujeres embarazadas hacia la vacuna COVID-19 y los factores que afectan a sus conocimientos y actitudes.

## Métodos

### Tipo de estudio

Esta investigación es un estudio descriptivo, transversal y analítico. Se basa en las directrices del *Strengthening the Reporting of Observational Studies in Epidemiology* (STROBE).

### Lugar y población del estudio

El estudio se llevó a cabo de enero a abril de 2022 en el norte de Chipre, que es una isla de 9.251 km^2^ en el Mediterráneo donde el turismo está muy extendido y hay muchos estudiantes matriculados en las 22 universidades. Por estos motivos, la población estudiantil, tanto académica como profesional, es bastante elevada. Además, Chipre del Norte es una isla con una tasa de natalidad muy baja y, según datos oficiales, el número total de nacimientos en el país en 2012 fue de 3.614^(24)^. En esta cifra también se incluyen los nacimientos de mujeres que tienen la condición de inmigrantes por motivos educativos y laborales.

Se incluyeron en el estudio las mujeres embarazadas mayores de 18 años que participaron voluntariamente en el estudio, independientemente de su edad gestacional. Los datos se recogieron por Internet para evitar el riesgo de infección debido a la pandemia. Las encuestas fueron diseñadas por los investigadores utilizando *Google Forms*. El investigador compartió el enlace al formulario de *Google* creado en plataformas de medios sociales (*WhatsApp*, *Messenger*, *Facebook* e *Instagram*). La primera página del cuestionario en línea incluía información sobre la finalidad y el contenido del estudio y el formulario de consentimiento informado para la participación en el estudio. El formulario se diseñó de tal forma que los que aceptaron participar continuaron con el cuestionario.

### Selección de la muestra

El número de mujeres embarazadas que debían incluirse en la muestra se determinó mediante el método de análisis de potencia. Se utilizaron las estadísticas de natalidad del país como referencia para analizar la potencia estadística^(23)^. El número de embarazadas necesario para el estudio fue de 365 con α = 0,05, 1-β = 0,80 y una tasa de error de 0,20. De las mujeres invitadas al estudio, 407 aceptaron participar y constituyeron el grupo de muestra del estudio. Una vez finalizado el estudio, se llevó a cabo un análisis de potencia *post-hoc* para determinar la adecuación del tamaño de la muestra. Según el análisis *post-hoc*, se determinó que la muestra total era suficiente con un tamaño del efecto de 0,92, una potencia del 99% y un margen de error de 0,05%.

### Criterios de inclusión

Se incluyeron en el estudio mujeres embarazadas de 18 años o más, sin barreras de comunicación (no hablantes de turco, problemas de salud mental, etc.), alfabetizadas y con conocimientos digitales básicos.

### Criterios de exclusión

Se excluyeron las embarazadas con algún factor de riesgo durante el embarazo (por ejemplo, preeclampsia, retraso del crecimiento intrauterino, rotura prematura de membranas, diabetes gestacional, etc.) y las embarazadas con algún problema diagnosticado relacionado con la salud del feto (por ejemplo, anomalías fetales y retraso del crecimiento intrauterino).

### Instrumentos de recogida de datos

Los primeros 14 ítems del formulario de 27 preguntas, preparado por el investigador después de revisar la bibliografía, se categorizaron para determinar las características sociodemográficas de las participantes^(20,24)^. Trece preguntas incluían el estado de vacunación de las participantes, sus opiniones sobre la vacunación con COVID-19 durante el embarazo y las fuentes de información sobre la vacuna^(1,7,24-25)^.

#### Escala de conocimientos y actitudes hacia la vacuna COVID-19

La escala, desarrollada en 2021 para evaluar el conocimiento y la actitud de los individuos hacia la vacuna COVID-19, es una escala tipo Likert de cinco puntos que consta de 16 preguntas^(26)^. La escala no tiene punto de corte. La elevada puntuación media obtenida en cada subescala indica que las percepciones relativas a la gravedad, el obstáculo y el beneficio de la subescala son elevadas. La escala consta de un total de tres subescalas: “Percepción de la gravedad de la enfermedad COVID-19” (5 ítems), “Percepción de los obstáculos de la vacuna” (7 ítems) y “Percepción del beneficio de la vacuna” (4 ítems). Al puntuar la escala, cada subescala se puntúa por separado. Mientras que las puntuaciones altas en las subescalas de gravedad y beneficio indican una actitud positiva hacia COVID-19, una puntuación alta en la subescala de obstáculos indica una actitud negativa hacia COVID-19. El coeficiente alfa de Cronbach de la escala utilizada en nuestro estudio se calculó en 0,83.

### Análisis de los datos

En el estudio, las características sociodemográficas y el estado de vacunación de las embarazadas, así como su distribución en función de algunas características relacionadas con la vacuna COVID-19, se determinaron mediante un análisis de frecuencias. Se proporcionaron estadísticas descriptivas relativas a las puntuaciones de la Escala de Conocimientos y Actitudes hacia la Vacuna COVID-19 de las embarazadas y se examinó la conformidad con la distribución normal con la prueba de Kolmogorov-Smirnov. En el estudio se utilizaron pruebas de hipótesis no paramétricas, como las pruebas U de Mann-Whitney y H de Kruskal-Wallis, porque las puntuaciones de la Escala de Conocimientos y Actitudes con respecto a la vacuna COVID-19 de las embarazadas no mostraban una distribución normal.

### Aspectos éticos

Este estudio fue aprobado por la Junta de Revisión Institucional de la Universidad (nº 2021/98-1444). Antes de iniciar la investigación, se obtuvo el consentimiento de todos los participantes y se garantizó la confidencialidad de la información personal. El estudio se realizó de acuerdo con los principios éticos de la Declaración de Helsinki.

## Resultados

La Tabla 1 presenta las características sociodemográficas del estudio realizado con la participación de 407 embarazadas. La edad media de las embarazadas participantes en el estudio fue de 27,61±4,73 años. Se determinó que el 27,27% de las participantes se encontraban en el primer trimestre de gestación, el 40,05% en el segundo y el 32,68% en el tercero. El 62,41% estaban en su primer embarazo, el 28,99% en el segundo y el 28,26% ya tenían un hijo.

Se determinó que el 13,51% de las embarazadas habían terminado la enseñanza secundaria, el 18,43% el bachillerato y el 52,83% la enseñanza superior. Alrededor de dos tercios, el 75,43%, tenían unos ingresos equivalentes a sus gastos, el 71,74% estaban empleadas y el 92,38% tenían una familia nuclear.

Este estudio descubrió que, aunque las puntuaciones de las subescalas de gravedad (*p* = 0,001) y beneficio (*p* = 0,001) de las mujeres embarazadas que habían completado la educación primaria eran más bajas (*p* = 0,001), las puntuaciones de las subescalas de barrera eran más altas (*p* = 0,001). Además, las embarazadas con un mayor nivel educativo tenían opiniones más positivas sobre la vacuna COVID-19 durante el embarazo (*p* ˂ 0,05).

Las puntuaciones de las subescalas de gravedad (*p* = 0,001) y beneficio (*p* = 0.001) fueron significativamente más altas, y las puntuaciones de la subescala de barreras (*p* = 0,001) fueron más bajas en las embarazadas del primer trimestre en comparación con las del segundo y tercer trimestre.

**Tabla 1 -:** Distribución de las mujeres embarazadas según sus características sociodemográficas y obstétricas (n = 407). Nicosia, Chipre, 2022

**Características sociodemográficas y obstétricas**	**n** ^*^	**%** ^†^
**Grupo de edad** ( **media 27,61±4,73 años)**		
24 años o menos	85	20,88
25-28 años	172	42,26
29 años o más	150	36,86
**Formación educativa**		
Educación primaria	27	6,63
Educación secundaria	55	13,51
Educación pré-universitaria	75	18,43
Educación universitaria	215	52,83
Posgrado	35	8,60
**Nivel de ingresos**		
Ingresos inferiores a los gastos	68	16,71
Ingresos iguales a los gastos	307	75,43
Ingresos superiores a los gastos	32	7,86
**Empleado**		
Sí	292	71,74
No	115	28,26
**Tipo de empleo**		
A tiempo completo	229	56,27
Tiempo parcial	178	43,73
**Tipo de familia**		
Familia nuclear	376	92,38
Familia extensa	31	7,62
**Duración del matrimonio**		
0-3 años	216	53,07
4-10 años	136	33,42
11 años o más	55	13,51
**Edad gestacional**		
1 ^er^ trimestre	111	27,27
2º trimestre	163	40,05
3 ^er^ trimestre	133	32,68
**Embarazo**		
Primer	254	62,41
Segundo	118	28,99
Tercero o más	35	8,60
**Número de hijos vivos**		
Ninguno	257	63,14
Uno	115	28,26
Dos o más	35	8,60

^*^n = Número de casos; ^†^% = Porcentaje

Se determinó que el 89,43% de las embarazadas incluidas en el estudio no se había vacunado contra el tétanos y el 100% no se había vacunado contra la gripe (influenza). Más de la mitad, el 51,84%, había recibido la vacuna COVID-19 antes del embarazo, el 36,12% tenía opiniones positivas sobre la vacunación COVID-19 durante el embarazo, mientras que el 63,88% tenía opiniones negativas. El 61,90% de las embarazadas que tenían una opinión positiva creían que la vacuna protege contra el virus COVID-19 durante el embarazo y el 75% de las que tenían una opinión negativa creían que la vacuna podría dañar al feto. Se observó que el 83,29% de las embarazadas recibieron información sobre la vacuna COVID-19 durante el embarazo y el 60,69% dijeron tener conocimientos suficientes sobre la vacuna COVID-19. La mayoría de ellas, el 86,73% de las embarazadas, dijo haber obtenido información sobre la vacuna COVID-19 de un médico, el 45,45% de las redes sociales, el 55,04% de expertos en televisión o Internet y el 28,99% de enfermeras (Tabla 2).

**Tabla 2 -:** Distribución de las embarazadas según su estado de vacunación y algunas características relacionadas con la COVID-19 (n = 407). Nicosia, Chipre, 2022

**Estado de vacunación y características relacionadas con COVID-19**	**n** ^*^	**%** ^†^
**Está vacunado contra el tétanos**		
Si	43	10,57
No	364	89,43
**Ha sido vacunado contra la influenza**		
No	407	100,00
**Presta atención a la higiene de las manos**		
Si	407	100,00
**Presta atención al distanciamiento social**		
Si	407	100,00
**Presta atención al uso de mascarilla**		
Si	407	100,00
**Contrajo COVID-19**		
Si	78	19,16
No	329	80,84
**Contrajo COVID-19 durante el embarazo**		
Si	52	12,78
No	355	87,22
**Situación personal respecto a la vacuna COVID-19**		
Recibí la vacuna COVID-19 antes del embarazo	211	51,84
Recibí la vacuna COVID-19 antes y durante el embarazo	32	7,86
Aún no me he vacunado y tengo intención de vacunarme durante el embarazo	41	10,07
Tengo intención de vacunarme después del embarazo	32	7,86
Tengo intención de vacunarme después de la lactancia	40	9,83
No tengo intención de recibir la vacuna COVID-19	51	12,53
**Opinión sobre recibir la vacuna COVID-19 durante el embarazo**		
Negativa	260	63,88
Positiva	147	36.12
**Motivo de la opinión positiva (n=147)**		
Creo que la vacuna me protegerá frente a COVID-19 durante el embarazo	91	61,90
Creo que la vacuna protegerá a mi bebé de COVID-19 durante el embarazo	56	38,10
**Motivo para la opinión negativa (n=260)**		
Creo que la vacuna perjudicaría a mi bebé	195	75,00
Creo que la vacuna supone un riesgo para la vida de mi bebé	123	47,31
Creo que la vacuna supone un riesgo para el desarrollo mental de mi bebé	107	41,15
**Recibió información sobre la vacuna COVID-19 durante el embarazo**		
Si	339	83,29
No	68	16,71
**Tenía conocimientos suficientes sobre la vacuna COVID-19**		
Si	247	60,69
No	160	39,31
**Qué fuentes de información sobre COVID-19 consultó**		
Médico	353	86,73
Redes sociales	185	45,45
Especialistas a través de TV/Internet	224	55,04
Enfermeras	118	28,99
Familiares y amigos	63	15,48

^*^n = Número de respondientes; ^†^% = Porcentaje

Las puntuaciones medias de las subescalas de gravedad y beneficio de las mujeres que se vacunaron antes del embarazo, que se vacunaron antes y durante el embarazo, las que aún no se han vacunado y las que tienen previsto vacunarse durante el embarazo fueron significativamente más altas, y las puntuaciones medias de las subescalas de barrera fueron significativamente más altas (*p* = 0,001). Las puntuaciones medias de las subescalas de gravedad y beneficio de quienes tenían una opinión positiva sobre la recepción de la vacuna COVID-19 durante el embarazo fueron superiores a las de quienes tenían una opinión negativa (*p* = 0,001). Se determinó que las que recibieron información sobre la vacuna COVID-19 durante el embarazo tenían puntuaciones medias más altas en la subescala de gravedad (*p* = 0,018). Se determinó que las que habían recibido información sobre la vacuna COVID-19 durante el embarazo y creían tener suficiente información tenían puntuaciones medias más altas en las subescalas de gravedad y competencia y puntuaciones medias más bajas en las subescalas de barrera (*p* < 0,05) (Tabla 3).

**Tabla 3 -:** Comparación de las puntuaciones medias de las subescalas de gravedad, barrera y beneficio de la escala de conocimientos y actitudes hacia la vacuna COVID-19 en función del estado de vacunación y de algunas características relacionadas con COVID-19 (n = 407). Nicosia, Chipre, 2022

**Estado de vacunación y características relacionadas con la COVID-19**	**Gravedad**			**Barrera**			**Beneficio**		
	**x̄** *	**DE** ^†^	p ^‡^	**x̄** *	**DE** ^†^	p ^‡^	x̄*	**DE** ^†^	p ^‡^
**Contrajo la infección por COVID-19**
Si	16,00	4,20	0,062	22,36	3,84	0,886	12,44	383	0,019
No	18,01	4,66		22,92	5,53		11,93	3,83	
**Contrajo infección por COVID-19 durante el embarazo**									
Si	15,00	4,85	0,003	23,54	4,24	0,029	11,15	4,13	0,224
No	18,01	4,48		22,71	5,38		12,16	3,77	
**Situación personal respecto a la vacuna de COVID-19**									
Ya vacunada antes del embarazo	18,97	3,95	0,001	22,42	4,43	0,001	13,00	3,21	0,001
Planea vacunarse antes y durante el embarazo	20,25	2,95		18,88	5,41		15,88	2,46	
Planea vacunarse durante el embarazo, pero aún no se ha vacunado	19,83	4,54		19,15	2,02		13,61	1,51	
Planea vacunarse después del embarazo	17,50	1,97		22,00	3,93		12,50	1,97	
Planea vacunarse después de la lactancia	13,50	4,56		23,00	5,06		9,50	3,54	
Nunca ha pensado en vacunarse contra COVID-19	11,96	2,47		30,25	3,45		6,00	0,00	
**Opinión sobre recibir la vacuna COVID-19 durante el embarazo**									
Negativa	16,15	4,47	0,001	24,32	5,62	0,001	10,77	4,10	0,001
Positiva	20,22	3,70		20,16	3,07		14,26	1,74	
**Obtuvo información sobre la vacuna COVID-19 durante el embarazo**									
Si	17,87	4,93	0,018	22,65	4,94	0,500	12,14	3,81	0,443
No	16,38	2,36		23,62	6,59		11,50	3,92	
**Cree que tiene suficiente información sobre la vacuna COVID-19**									
Si	18,36	4,77	0,001	22,17	5,30	0,031	12,30	4,05	0,031
No	16,48	4,18		23,81	5,03		11,61	3,42	

^*^x- = Media; ^†^DE = Desviación estándar; ^‡^ *p* < 0,05, estimado mediante la prueba de Wald

## Discusión

Los resultados de este estudio revelan que, si bien las mujeres tienen actitudes positivas hacia la vacunación cuando no están embarazadas, no ocurre lo mismo durante el embarazo. El hecho de que la tasa de vacunación sistemática contra el tétanos durante el embarazo sea tan baja sugiere que la cuestión de la vacunación durante el embarazo puede haberse pasado por alto. Diferentes estudios también han informado de que la vacuna contra la gripe estacional no es habitual entre las embarazadas y a menudo no es aceptada por ellas^(27-30)^. Además, se descubrió que el 63,88% de las embarazadas tenían una opinión negativa sobre la vacunación contra la COVID-19 durante el embarazo, ya que el 75% de ellas creía que la vacuna podía dañar al feto y el 47,31% pensaba que la vacuna podía suponer un riesgo para la vida del bebé. Algunos estudios han revelado que las embarazadas consideran que las vacunas podrían perjudicarlas a ellas mismas o al feto, causar infertilidad y que el riesgo de contraer la COVID-19 era bajo durante el embarazo^(7,31)^; en particular, las percepciones sobre los efectos de la vacuna en el feto pueden afectar negativamente a la actitud de las embarazadas hacia la vacuna. En un estudio, a diferencia del nuestro, la tasa de vacunación durante el embarazo era elevada, y se concluyó que, a pesar de las reservas sobre la vacuna COVID-19, una actitud decidida y positiva hacia las vacunas en general aumentaba la aceptación de la vacuna contra COVID-19^(10)^.

Se encontró que las puntuaciones de las subescalas de gravedad y beneficio de las mujeres embarazadas en el primer trimestre eran más altas. En la literatura, se ha demostrado que se obtuvieron diferentes resultados cuando se evaluó la aceptación de la vacuna según la edad gestacional. Un estudio en Turquía encontró que la aceptación de la vacuna fue mayor en el primer trimestre^(24)^. Un estudio realizado en China indicó que las mujeres embarazadas en el segundo y tercer trimestre estaban más dispuestas a recibir la vacuna contra la COVID-19 que las del primer trimestre^(32)^.

Se observó que las embarazadas con un alto nivel de educación tenían una perspectiva positiva de la vacuna contra COVID-19, las puntuaciones en la subescala de gravedad y beneficios de la vacuna COVID-19 eran más altas y las puntuaciones en la subescala de barreras eran más bajas. Los estudios realizados tanto en mujeres embarazadas como en la población general han demostrado que un alto nivel educativo influye positivamente en la aceptación de la vacuna^(25,30,33)^. Además, las puntuaciones en las subescalas de gravedad y beneficio de las embarazadas que dijeron tener suficiente información sobre COVID-19 fueron más altas, y las puntuaciones en la subescala de barrera fueron más bajas. Esto demuestra que educar a las mujeres embarazadas sobre COVID-19 afectó positivamente a su actitud hacia la vacunación. Diferentes estudios respaldan los resultados de este estudio^(10,25,34)^.

Los resultados de la bibliografía también muestran que las mujeres embarazadas deberían disponer de información adecuada sobre la eficacia y la seguridad de la vacuna contra COVID-19^(35-36)^. Se observó que las embarazadas concedían gran importancia a la atención a la higiene de las manos, el uso de mascarillas y el distanciamiento social, basándose en medidas preventivas distintas de la vacunación. Del mismo modo, en un estudio se observó que las embarazadas prestaban gran atención al uso de mascarillas, el lavado de manos y el distanciamiento social^(25)^. Estos resultados muestran que las embarazadas prestan atención a otros métodos preventivos de la COVID-19.

Aunque el 39,31% de las embarazadas afirmaron no tener suficientes conocimientos sobre la vacuna contra COVID-19, dijeron que obtenían información principalmente de los médicos (86,73%), seguidos de los expertos a través de la televisión e Internet (55,04%). En un estudio, la principal fuente de información fueron los medios de comunicación^(24)^. Especialmente durante los periodos de encierro, Internet se utilizó ampliamente como fuente de información de los medios de comunicación. Sin embargo, estas fuentes también pueden conducir a la desinformación. Dado que la desinformación sigue extendiéndose, las recomendaciones de los profesionales sanitarios como fuente fiable de información para las embarazadas son cruciales^(37)^. Un ensayo controlado aleatorizado que evaluó las actitudes de las embarazadas hacia la vacunación mediante el envío de mensajes de texto sobre la vacuna contra COVID-19 demostró tener un efecto positivo en las actitudes de las embarazadas hacia la vacunación^(38)^. La tasa de mujeres embarazadas que dicen haber recibido información sobre la vacuna contra COVID-19 por parte de las enfermeras es de aproximadamente el 30%. La tasa de mujeres embarazadas que dicen haber recibido información sobre la vacuna contra COVID-19 por parte de las enfermeras es de aproximadamente el 30%. Las enfermeras trabajan en instituciones sanitarias donde se prestan servicios de salud preventiva, y también desempeñan funciones de educación sanitaria. Por lo tanto, la proporción de mujeres embarazadas que recibieron información sobre la vacuna contra COVID-19 de las enfermeras es intrigante.

Este estudio es importante para demostrar que las actitudes negativas hacia las vacunas durante el embarazo pueden ser un enfoque similar en términos de aceptación de nuevas vacunas. Deberían realizarse estudios de educación y concienciación sobre la aceptación y las actitudes hacia las vacunas durante el embarazo, especialmente fuera de los periodos epidémicos, y debería garantizarse la continuidad de estos estudios. Además, nuestro estudio revela que las enfermeras deberían utilizar su papel de educadoras para llegar a más mujeres con información sobre las vacunas durante el embarazo.

El idioma del estudio es el turco, por lo que sus resultados no pueden generalizarse a toda la isla. Como este estudio se realizó en línea y la encuesta se compartió a través de las redes sociales, se incluyó en él a mujeres embarazadas con conexión a Internet y usuarias de las redes sociales. Otra limitación es que la respuesta a las preguntas depende de la subjetividad de las personas, ya que los datos se recogieron en línea.

## Conclusión

En el presente estudio, se determinó que las mujeres embarazadas tenían una actitud negativa hacia la vacunación durante el embarazo y esta actitud negativa continuó hacia las vacunas contra COVID-19. La razón más destacada de la actitud negativa hacia las vacunas contra COVID-19 era la preocupación de que pudieran afectar negativamente al embarazo y dañar al feto. Cuanto mayor era el nivel educativo de las embarazadas, más positivas eran sus actitudes hacia la vacunación contra COVID-19 durante el embarazo. Además, la obtención de información suficiente sobre la vacuna contra COVID-19 afectaba a las actitudes de las embarazadas hacia la vacunación. Según estos resultados, es importante ofrecer formación a las embarazadas para protegerlas en el grupo desfavorecido y garantizar la sostenibilidad de las vacunas contra COVID-19. Facilitar información sobre la vacuna contra COVID-19 y sus efectos no bastará por sí solo para prevenir nuevos brotes.

Resultó que la gran mayoría de las personas que tienen una actitud negativa hacia la vacuna contra COVID-19 creen que la vacuna perjudicará su embarazo. Además, el hecho de que otras vacunas tengan tasas muy bajas durante el embarazo revela que tienen una actitud negativa hacia la vacunación durante el embarazo. Por lo tanto, no debe ignorarse la información sobre la importancia de la vacunación durante el embarazo para proteger la salud del feto y de la madre. También se recomienda realizar estudios cualitativos, que incluyan entrevistas en profundidad, para determinar las causas de las preocupaciones de las mujeres sobre la vacunación.
